# Supplementary material for: Persistence as a Constituent of a Biocontrol Mechanism (Competition for Nutrients and Niches) in Pseudomonas putida PCL1760
Source: Microorganisms. 2022 Dec 21;11(1):19. doi: 10.3390/microorganisms11010019 (PMC9867257; doi:10.3390/microorganisms11010019)
Supplement: Supplementary file 1 [file microorganisms-11-00019-s001.zip › microorganisms-2087822-supplementary.pdf]

Supplementary Materials

Persistence as a constituent of biocontrol mechanism (competition for nutrients and niches) in *Pseudomonas putida* PCL1760

Aynur Kamilevich Miftakhov, Roderic Gilles Claret Diabankana, Mikhail Frolov, Marat Miratovich Yusupov, Shamil Zavdatovich Validov and Daniel Mawuena Afordoanyi

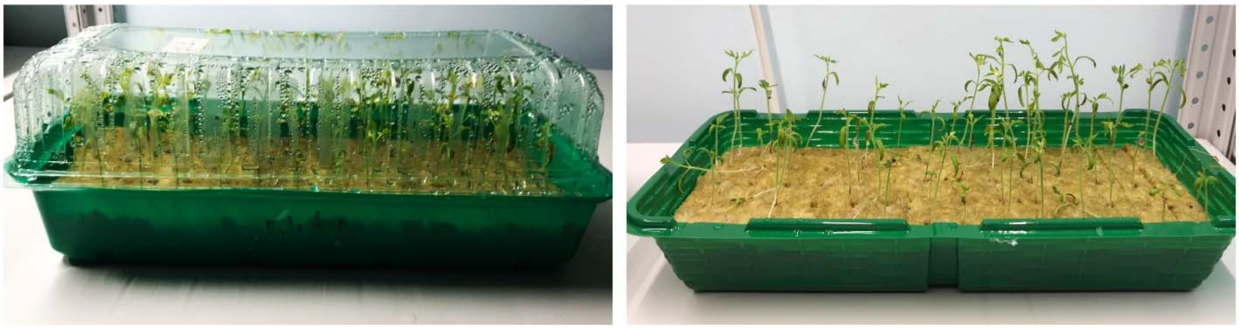

Figure S1. Biocontrol experiment. Tomato plants growth three weeks after seed inoculation in pots containing rockwool presoaked in a spore suspension of *Forl* ZUM2407.

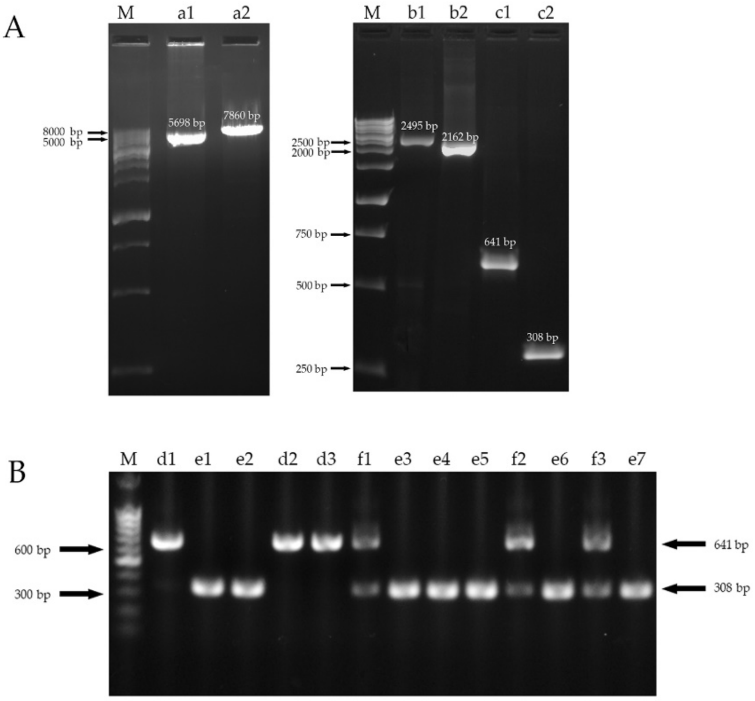

Figure S2. Electrophoresis gel diagram of confirmation tests for construction and acquisition of PCL1760 mutant SB9. Gel data confirmed the stages of genetic construction and the acquisition of the mutant (**A**); (**B**) PCR-fragment performed on different colonies grown on the nutrient medium. **M**-1 Kb DNA Ladder; **a1**-pk18mobsacB (linear); **a2-**pk18mobsacB: (flΔ*rsf*S); **b1-**fl-*rsf*S; **b2** -flΔ*rsf*S); **c1**- Fragment obtained after performing PCR with test-rsfS primers on *P.putida* PCL1760 chromosomal DNA; **c2**- Fragment obtained after performing PCR with test-rsfS primers on pk18mobsacB: (flΔrsfS); **d1-d3**- *P. putida* PCL1760; e**1-e7**- *P. putida* SB9; **f1-f3**- pk18mobsacB: (flΔrsfS).
